# Impact of an exercise and nutrition program on caregiver time with residents in institutional care—A secondary analysis

**DOI:** 10.1002/alz.71198

**Published:** 2026-02-14

**Authors:** Anders Wimo, Tommy Cederholm, Gerd Faxén Irving, Erika Franzén, Helena Grönstedt, Åke Seiger, Sofia Vikström, Anne‐Marie Boström

**Affiliations:** ^1^ Department of Neurobiology Care Sciences and Society Center for Alzheimer Research Division of Neurogeriatrics BioClinicum Karolinska Institute Solna Sweden; ^2^ Department of Public Health and Caring Sciences Uppsala University Uppsala Sweden; ^3^ Theme Inflammation & Aging Karolinska University Hospital Stockholm Sweden; ^4^ Department of Neurobiology, Care Sciences and Society, Division of Clinical Geriatrics Karolinska Institute Stockholm Sweden; ^5^ Department of Neurobiology Care Sciences and Society Division of Physiotherapy Karolinska Institute Stockholm Sweden; ^6^ Women's Health and Allied Health Professionals Theme, Medical Unit Health Professionals Karolinska University Hospital Stockholm Sweden; ^7^ Stockholms Sjukhem R&D Unit Stockholm Sweden; ^8^ Department of Neurobiology Care Sciences and Society Division of Clinical Geriatrics Karolinska Institute Stockholm Sweden; ^9^ Department of Neurobiology Care Sciences and Society Division of Occupational Therapy Karolinska Institute Stockholm Sweden

**Keywords:** caregiver time, dementia, health promotion, nursing homes, nutrition, older adults, OPEN study, physical activity, protein supplementation, sit‐to‐stand exercise, staff

## Abstract

**INTRODUCTION:**

Residents in long‐term institutional care (LTIC) settings are at risk of malnutrition, sarcopenia, and frailty. In the Older Persons Exercise and Nutrition (OPEN) randomized study, the impact of exercise and nutrition was analyzed. This secondary analysis focused on caregiver time (CGT).

**METHODS:**

The 3‐month intervention included repeated sit‐to‐stand exercises and two protein‐enriched supplements daily. CGT was assessed in both dementia and somatic units using the Resource Utilization in Dementia instrument. Non‐linear methods were applied due to skewed data.

**RESULTS:**

The sample consisted of 102 persons (intervention group [IG] *n* = 52, control group [CG] *n* = 50). CGT in the IG was significantly lower at follow‐up, adjusted for baseline CGT, in the dementia units, that is, 55 min/day and resident, compared to 83 min/day in the CG (odds ratio 0.668 [0.473–0.945]; *p* = 0.022).

**DISCUSSION:**

A structured exercise and nutrition program was associated with reduced CGT in the dementia, but not the somatic, LTIC units.

**CLINICAL TRIAL REGISTRATION:**

This study has been registered with the number protocol of ClinicalTrials.gov Identifier: NCT02702037.

## Highlights

- Residents in long‐term institutional care settings risk malnutrition, sarcopenia and frailty.
- Older Persons Exercise and Nutrition (OPEN) is a program including exercise and nutrition.
- Caregiver time (CGT) is a heavy cost‐driver in long‐term institutional care.
- The OPEN‐program reduced CGT in the dementia units.

## 1 BACKGROUND

Long‐term institutional care (LTIC), such as in nursing homes, is an important part of the care infrastructure for older people in many high‐income countries. However, demographic changes with an increasing number of very old people, combined with a strained economy in the welfare systems, jeopardize the quality of care in such settings. The residents have increasing care needs, including complex medical conditions such as undernourishment and physical inactivity, contributing to sarcopenia and frailty. Since staff resources are frequently insufficient in relation to the residents’ care needs,^1^, ^2^ it is important to develop care strategies in LTIC that promote effective use of resources, such as caregiver time (CGT).

In health economic analyses of the care of older people, costs for people residing in LTIC are usually standardized and based on mean data at group level, not on individual characteristics in terms of time for various resource use and care activities. In LTIC, CGT is the most important cost‐driver and includes several care domains, such as support with basic activities of daily living (ADL) instrumental ADL, and supervision/surveillance activities. However, there are differences in time use and workload in LTICs, mainly related to the levels of functional and cognitive status of the residents. There are few studies focusing on CGT in LTIC and most are descriptive.^3^, ^4^, ^5^, ^6^, ^7^, ^8^, ^9^, ^10^, ^11^, ^12^, ^13^ For example, in a Swedish study, the CGT was about 120 min/day,^3^ and in a German study, it was a bit lower, about 70 min per day.^5^

Functional and cognitive status has implications for the possibilities for interventions that may affect the CGT.^3^, ^4^ We have found two randomized controlled trials in LTIC where CGT was analyzed, both concerning managing dementia symptoms.^14^, ^15^ However, in Livingstone et al.’s study, CGT in terms of minutes or hours per day or week was not presented in detail; it was instead included in the resulting cost of the care.^14^ In the study by Luttenberger et al., positive effects were shown on symptoms but not on CGT, which could be explained by the intervention being performed by research staff and not the nursing home staff.^15^ The authors discussed that, even if the residents improved in ADL capacity, the nursing home staff may have continued to support ADL routinely, even if help was not needed, due to lack of knowledge, highlighting the importance of involving the staff in interventions.

The Older Persons Exercise and Nutrition (OPEN) study aimed to investigate the impact on physical function, nutritional status, health‐related quality of life and resource use (see details in the OPEN study protocol paper^16^) of sit‐to‐stand exercises (STS) combined with a protein‐rich oral nutritional supplement (ONS) integrated into the older persons’ daily care. The OPEN study results have been reported in several publications.^17^, ^18^, ^19^, ^20^ Perhaps the most important finding was that post hoc per protocol analysis indicated that chair‐rise capacity, i.e., the primary outcome, was significantly better among the 40% of the participants who could adhere to the intervention program to a greater extent compared to controls and low‐adherers. Body weight and muscle mass were also substantially improved. Qualitative interview studies with the older participants and the supporting staff corroborated the partially positive outcome of the intervention by, for example, highlighting ease of use.^19^, ^21^

In this secondary analysis of the OPEN study, we hypothesized that the combined intervention would result in a reduction in CGT in support of basic ADL at the intervention units, as a possible result of improvements in physical and cognitive function, as well as in the parameters indicating nutritional status. Furthermore, we analyzed whether there were any differences between units assigned for somatic and dementia care, respectively.

## 2 METHODS

### 2.1 Study design, setting, and participants

The OPEN study is a two‐arm cluster‐randomized controlled trial performed in eight LTICs including 62 units in two municipalities in the Stockholm area completed in 2016–2018, with altogether 495 residents. A cluster was defined as a group of 1‐3 LTIC units, which were randomized according to a computer‐based sample list into two groups, an intervention group (IG) and a control group (CG). Each cluster included units designated for either dementia care or somatic care. For practical and logistic reasons, a cluster‐design was used for randomization, whereas statistical analyses were based on individual outcomes. Older persons with various dementia diagnoses resided in the dementia units, while older persons with limitations mainly in physical functioning dominated in the somatic units. The study was approved by the Regional Ethical Review Board in Stockholm, Dno. 2013/1659‐31/2, 2015/1994‐32 and 2016/1223‐32.

**RESEARCH IN CONTEXT**

1. **Systematic review**: Although care in long‐term care institutional (LTIC) settings has been frequently studied from various viewpoints, few studies focus on caregiver time (CGT), most are descriptive, and very few are randomized trials.
2. **Interpretation**: A structured exercise and nutrition intervention, the Older Persons Exercise and Nutrition program (OPEN), was in a randomized controlled trial associated with reduced CGT at the dementia units, but not at the somatic LTIC units.
3. **Future directions**: Since results are based on a secondary analysis, further studies with a focus on different aspects of CGT as well as a more comprehensive assessment of resource use, costs and outcomes are needed.

**FIGURE 1:**
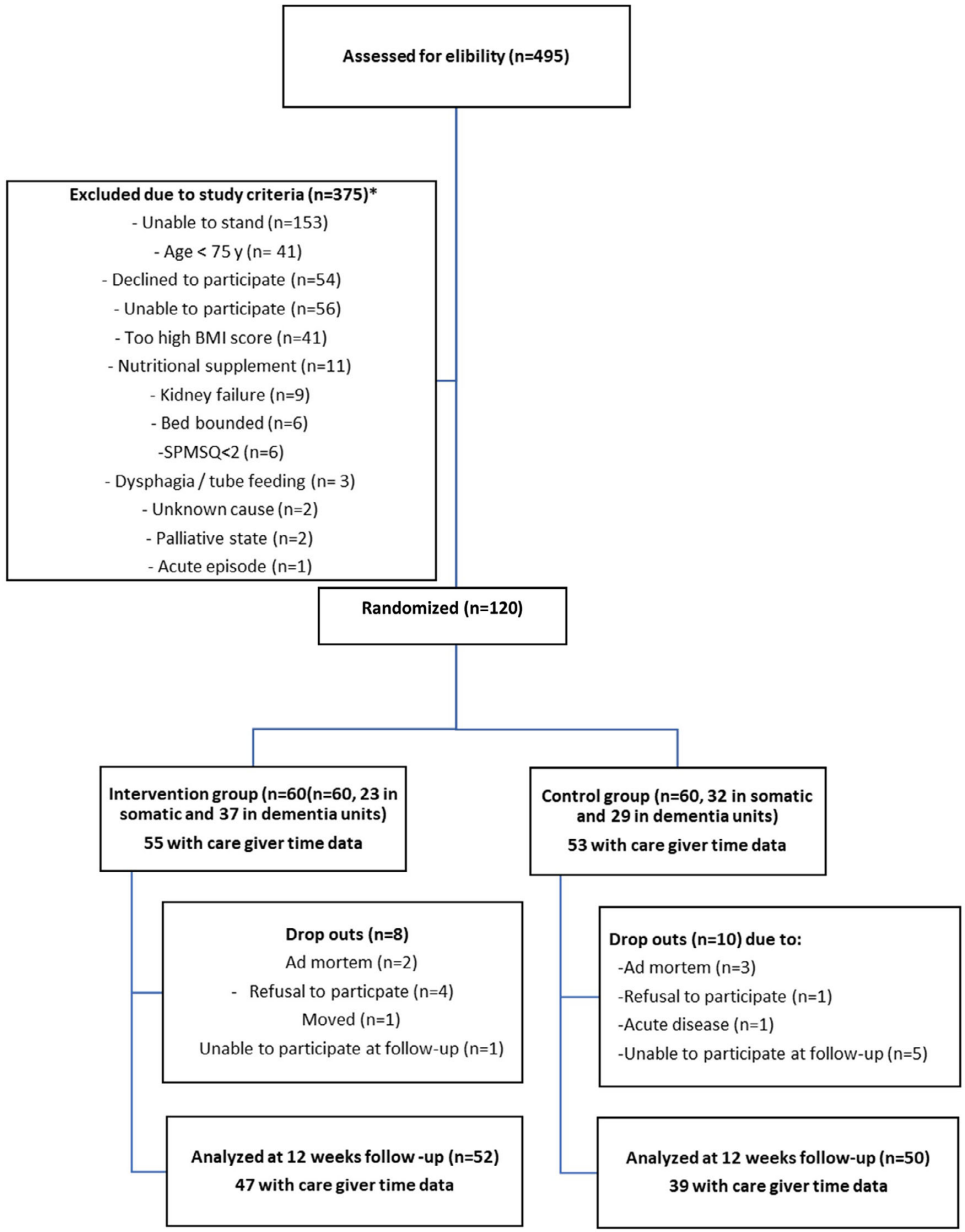
Flow chart of participants through the trial. ^*^The same resident could be registered for more than one exclusion criteria. SPMSQ = Short Portable Mental Status Questionnaire.

LTIC residents aged ≥75 years and able to stand with or without support were invited to participate. Verbal informed consent was given before study inclusion. The exclusion criteria were: body mass index (BMI) > 30 kg/m^2^, treatment with protein‐rich oral supplements, severe dysphagia, tube feeding, being bedridden, severe kidney disease, terminal stage of life and lack of informed consent. The power analysis indicated a need for 120 participants (power 0.8, *p* ≤ 0.05) to reveal a relevant difference in the chair‐stand test (CST), the primary outcome. Altogether, 174 out of 495 residents were considered eligible for randomization (Figure 1), while 54 declined participation.^16^ Thus, 120 residents were randomized to either the IG (*n* = 60) or CG (*n* = 60). During the observation period, 18 residents dropped out, while 102 residents completed the study and were included in the statistical analysis (Figure 1).

### 2.2 Intervention

The intervention period lasted for 12 weeks and the participants in the IG performed the STS exercise, preferably four times per day, 7 days a week, integrated into their regular activities of daily living. The exercises were supported by staff when necessary. The participants were also offered an oral protein‐rich supplement (125 mL containing 18 g protein, corresponding to around a quarter of the recommended dietary intake of protein, and 300 kcal) twice a day, 7 days per week. The staff at the intervention units were informed about the purpose of the study and were trained in how to support and encourage participants as well as on how to document the occasions of STS and the amount of consumed ONS for each participant in flowcharts. The flowcharts were collected weekly to monitor the adherence to the protocol. The participants in the CG received standard care.

### 2.3 Measurements

#### 2.3.1 CGT

The Resource Utilization in Dementia instrument (RUD) was developed to quantify the use of resources, particularly in dementia care.^22^ RUD includes the time (minutes per day) the nursing staff spend assisting each resident with basic ADL, instrumental ADL, supervision, informal caregiver support in the same domains and in various living/care situations (at home or in different kinds of LTIC facilities). RUD also includes hospitalization days, number of visits to/by various types of healthcare and social care professionals. RUD has been extensively validated,^5^, ^8^, ^9^, ^23^, ^24^, ^25^ and the CGT estimates (both by staff and informal carers) have been validated vs. time measured by the clock.^24^ The instrument was administrated via interviews with staff responsible for care of the residents. In the present paper we focus on CGT in terms of support in basic ADLs.

#### 2.3.2 Physical function

The 30‐second CST was the primary outcome in the project^26^, ^27^, ^28^ on which the power calculations were thus based on. Dependence in ADL was assessed according to the Functional Independence Measure (FIM), which consists of 18 items measuring physical and cognitive functions.^29^ In this study, the FIM ADL items were used (score from 12 to 91 points), where higher scores indicate a higher level of independence.

#### 2.3.3 Nutritional status

The Mini Nutritional Assessment‐Short Form (MNA‐SF) was used to assess nutritional status.^30^ It includes six domains: decline in food intake, weight loss, mobility, physiological stress or acute disease, cognitive condition, and BMI or calf circumference, and yields scores from 0 to 14 points; 12–14 = normal nutritional status; 8–11 = at risk for malnutrition; 0–7 = malnourished. BMI was calculated (kg/m^2^).

#### 2.3.4 Other measurements

Demographic data on age and sex were collected from the residents’ patient records. At baseline, cognitive function was assessed using the Mini‐Mental State Examination (0–30 points),^31^ risk of sarcopenia using the SARC‐F questionnaire (0–10 points; ≥4 points = increased risk),^32^ and frailty using the FRAIL questionnaire (0–5 points; ≥3 points = frailty; 1–2 points = prefrail).^33^

#### 2.3.5 Statistical analysis

As indicated, statistical analyses were based on individuals and not clusters. Demographic and clinical data were reported as mean and standard deviation for continuous variables, and number and percentage for categorical data. Since data on CGT (and other resource use) are usually skewed,^34^ non‐linear analysis was applied. A non‐linear regression analysis of the baseline data (logarithmized) was applied to analyze which covariates best predicted CGT. For the analysis of CGT at follow‐up comparing the intervention and control groups, a generalized linear model (GLM) with a gamma distribution with log link was applied with odds ratios (ORs) and corresponding 95% confidence intervals (CIs). All statistical analyses were performed in IBM® SPSS® statistics, version 28.0.1.1 (14).

#### 2.3.6 Sensitivity analysis

The base case analysis is based on observed cases (OC), since other published studies from the OPEN study are based on OC and we wish to facilitate comparisons. However, since it is recommended in intervention studies to base results on an intention‐to‐treat (ITT) analysis we have in a sensitivity analysis imputed missing data with the Expectation‐Maximization (EM) algorithm.^35^

## 3 RESULTS

The characteristics of the study sample are presented in Table 1. Of the 120 residents participating in the study, the mean age was 86 years, and the majority were women. There were no statistically significant baseline differences between the IG and the CG. However, there were differences in several domains between the dementia and the somatic units. The residents at the dementia units were older than those at the somatic units (*p* < 0.001), their cognitive status was more impaired (*p* < 0.001), they had better ADL performance (*p* = 0.007), were less frail (*p* < 0.001) and had less risk of sarcopenia (*p* < 0.001). There were no major differences between completers of the study and dropouts (Table 2). However, in the CG, there were more dropouts among residents with more severe conditions in FIM ADL items (*p* = 0.007) and sarcopenia (*p* = 0.018).

**TABLE 1:** Descriptive and comparative data for the study groups at baseline.

Parameter	Intervention group (IG)	Control group	*p*‐value between IG and CG
N	60	60	
Age (SD)	86.0 (5.1)	86.2 (5.7)	0.853
Gender (female %)	60%	58.3 %	0.500
Cognitive status (MMSE) (SD)	18.0 (5.7)	18.8 (6.3)	0.524
30‐sec chair‐stand test	5.9 (3.2)	6.0 (3.1)	0.816
ADL (FIM‐motor items (SD)	67.0 (20.4)	65.0 (21.1)	0.583
Sarcopenia (SARC‐F) (SD)	3.7 (2.99)	3.2 (2.77)	0.348
Frailty (FRAIL) (SD)	1.0 (1.19)	1.1 (1.17)	0.422
Nutritional status (MNA‐SF) (SD)	11.6 (1.9)	11.7 (1.8)	0.805
BMI (SD)	25.9 (3.8)	25.1 (3.7)	0.280
CGT (minutes per day) (SD)	91.8 (79.7)	94.9 (82.8)	0.844

	Intervention group		Control group		*p*‐value between IG and CG at dementia units	*p*‐value between IG and CG at somatic units	*p*‐value in dementia and somatic units overall
Parameter	Dementia units	Somatic units	Dementia units	Somatic units			
N	37	23	29	31			
Age (SD)	85.7 (5.0)	86.4 (5.3)	83.2 (4.4)	89.0 (5.3)	0.035	0.092	<0.001
Gender (female %)	64,9%	52,2%	58,6%	58,1%	0.395	0.438	0.294
Cognitive status (MMSE) (SD)	16.0 (5.3)	21.1 (4.9)	15.5 (4.9)	22.1 (5.6)	0.760	0.534	<0.001
30‐sec chair‐stand test	6.2 (3.4)	5.4 (2.7)	6.6 (3.3)	5.6 (2.8)	0.715	0.809	0.117
ADL (FIM‐motor items) (SD)	72.9 (16.8)	57.6 (22.4)	67.5 (18.2)	62.6 (23.6)	0.218	0.441	0.007
Sarcopenia (SARC‐F) (SD)	2.1 (1.9)	6.1 (2.7)	1.9 (2.6)	4.4 (2.3)	0.775	0.020	<0.001
Frailty (FRAIL) (SD)	0.5 (0.8)	1.7 (1.3)	0.7 (0.8)	1.5 (1.3)	0.285	0.609	<0.001
Nutritional status (MNAs‐SF) (SD)	11.3 (1.5)	12.0 (2.4)	11.4 (1.9)	11.9 (1.7)	0.845	0.845	0.054
BMI (SD)	26.1 (3.6)	25.6 (4.0)	25.8 (4.1)	24.5 (3.2)	0.765	0.290	0.159
CGT (minutes per day) (SD)	83.0 (72.0)	105.0 (90.2)	94.6 (89.8)	95.2 (77.2)	0.584	0.683	0.466

Abbreviations: ADL, activities of daily living; BMI, body mass index; CGT, caregiver time; FIM, the Functional Independence Measure; MMSE, Mini‐Mental State Examination; MNA‐SF, Mini Nutritional Assessment – Short Form; SD, standard deviation.

**TABLE 2:** Baseline data of completers and dropouts at follow‐up.

	Intervention group			Control group		
Parameter	Completers	Dropouts	*p*‐value	Completers	Dropouts	*p*‐value
N	52	8		50	10	
Age (SD)	85.8 (5.0)	87.0 (5.6)	0.554	85.9 (5.4)	87.8 (7.0)	0.361
Gender (female %)	65.4 %	25.0 %	0.050	58.0 %	60%	0.597
MMSE, 0–30 p (SD)	17.9 (6.0)	18.6 (1.5)	0.805	18.4 (5.9)	20.4 (8.0)	0.395
ADL (FIM‐motor items, 12–91) (SD)	67.3 (21.3)	65.1 (14.3)	0.779	68.2 (18.3)	48.8 (27.4)	0.007
Sarcopenia (SARC‐F, 0–10 p (SD))	3.5 (2.9)	4.8 (3.3)	0.269	2.7 (2.7)	5.0 (2.4)	0.018
Frailty (FRAIL, 0–5 p) (SD))	0.9 (1.1)	1.0 (1.4)	0.898	0.9 (1.0)	2.0 (1.2)	0.08
Nutritional status (MNA‐SF, 0–14 p) (SD)	11.6 (1.8)	11.5 (2.6)	0.897	11.8 (1.9)	11.2 81.1)	0.364
BMI, kg/m^2^ (SD)	25.6 (3.6)	28.3 (4.6)	0.101	25.3 (3.8)	24.5 (3.7)	0.559
CGT min (SD)	88.9 (71.0)	108.8 (124.3)	0.521	89.4 (83.4)	121.7 (78.8)	0.292

Abbreviations: ADL, activities of daily living; BMI, body mass index; CGT, caregiver time; FIM, the Functional Independence Measure; MMSE, Mini‐Mental State Examination; MNA‐SF, Mini Nutritional Assessment – Short Form; SD, standard deviation.

At baseline, the mean CGT was 92 min/day and resident in the IG and 95 in the CG (Table 1). The CGT at baseline varied to some extent between the somatic and dementia units, but the differences were not statistically significant. In the raw and unadjusted data, CGT decreased in the total sample during the follow‐up period (Table 3), but more in the IG than the CG. However, there were also differences between the dementia and somatic units in the IG. There was no or very low use of other resources in the RUD‐instrument such as hospitalization, visits to clinics, or emergency room visits, with no differences between the groups.

**TABLE 3:** CGT (minutes per day) at baseline and at follow up, unadjusted mean raw data.

Parameter	Baseline all	Baseline completers	Follow‐up	Mean change of completers
No. of participants	108	86	86	
Intervention group	91.8	88.9	73.0	−15.9
Control group	94.9	89.4	84.9	−4.5
All	93.3	89.2	78.4	−10.8
Intervention group: dementia units	83.0	77.2	59.1	−18.1
Control group: dementia units	94.6	94.8	97.5	2.7
Intervention group: somatic units	105.0	107.8	95.6	−12.2
Control group: somatic units	95.2	85.0	68.5	−16.5

Abbreviations: ADL, activities of daily living; BMI, body mass index; CGT, caregiver time;

In the non‐linear regression analysis, ADL and nutritional status (according to MNA‐SF) were the strongest predictors of CGT at baseline (Table 4). Thus, these factors were used as co‐variates in the GLM‐analysis (Table 5). There were no statistically significant differences in CGT at baseline between the IG and CG (when dementia and somatic units were combined), either at baseline or follow‐up; for example, 64 min/day in the IG and 76 min/day in the CG at baseline (OR 0.844 [0.655–1.087]; *p* = 0.188). However, at follow‐up, CGT was significantly lower in the dementia units (adjusted for baseline CGT) in those who underwent the intervention; that is, 55 min/day in the IG and 83 min/day in the CG (OR 0.668 [0.473–0.945]; *p* = 0.022).

**TABLE 4:** Stepwise regression analysis of CGT at baseline: first step and last step, dependent variable and co‐variates logged (Ln).

Parameter	B	SE	Beta	T	*p*‐value	Lower 95% CI for B	Upper 95% CI for B
First step							
(Constant)	6.639	9.107		0.729	0.473	−12.117	25.394
Frailty (FRAIL)	0.262	0.291	0.141	0.900	0.376	−0.337	0.861
Sarcopenia (SARC‐F)	0.099	0.250	0.071	0.397	0.694	−0.414	0.612
ADL (FIM motor items)	−0.704	0.304	−0.396	−2.318	0.029	−1.328	−0.080
Nutritional status (MNA‐SF)	−1.531	0.562	−0.416	−2.724	0.011	−2.687	−0.376
BMI	0.763	0.805	0.136	0.948	0.352	−0.891	2.418
Cognitive status (MMSE)	−0.506	0.378	−0.203	−1.337	0.193	−1.283	0.272
Final step							
(Constant)	11.236	1.347		8.341	<0.001	8.485	13.987
ADL (FIM motor items)	−1.000	0.239	−0.563	−4.190	<0.001	−1.487	−0.512
Nutritional status (MNA‐SF)	−1.112	0.495	−0.302	−2.248	0.032	−2.123	−0.102

Abbreviations: ADL, activities of daily living; BMI, body mass index; CI, confidence interval; FIM, the Functional Independence Measure; MMSE, Mini‐Mental State Examination; MNA‐SF, Mini Nutritional Assessment – Short Form.

**TABLE 5:** GLM analysis of CGT at follow‐up

Parameter	B	SE	Sig	OR	Lower 95% CI of OR	Upper 95% CI of OR	CGT at follow‐up	Lower 95% CI	Upper 95% CI
**All**									
(Intercept)	2.332	1.177	0.047	10.301	1.026	103.405			
Intervention group	−0.170	0.129	0.188	0.844	0.655	1.087	63.7	53.7	75.6
Control group	0						75.5	62.8	90.8
Age	0.025	0.013	0.044	1.026	1.001	1.051			
Gender	0.210	0.138	0.127	1.234	0.942	1.616			
ADL (FIM)	−0.016	0.004	<0.001	0.984	0.977	0.992			
Nutritional status (MNA‐SF)	0.006	0.038	0.867	1.006	0.935	1.084			
Baseline CGT	0.005	0.001	<0.001	1.005	1.003	1.007			
**Dementia units**									
(Intercept)	1.258	1.957	0.521	3.517	0.076	162.934			
Intervention group	−0.403	0.177	0.022	0.668	0.473	0.945	55.2	44.1	69.2
Control group	0						82.6	64.6	105.8
Age	0.043	0.020	0.034	1.043	1.003	1.085			
Gender	0.099	0.183	0.587	1.104	0.772	1.580			
ADL (FIM)	−0.015	0.006	0.007	0.985	0.974	0.996			
Nutritional status (MNA‐SF)	0.004	0.058	0.941	1.004	0.897	1.125			
Baseline CGT	0.004	0.001	0.005	1.004	1.001	1.007			
**Somatic units**									
(Intercept)	0.287	1.798	0.873	1.333	0.039	45.232			
Intervention group	0.142	0.213	0.505	1.152	0.759	1.748	75.2	57.5	98.3
Control group	0						65.2	49.0	86.9
Age	0.042	0.019	0.030	1.043	1.004	1.083			
Gender	0.389	0.236	0.100	1.475	0.929	2.342			
ADL (FIM)	−0.021	0.007	0.002	0.979	0.967	0.992			
Nutritional status (MNA‐SF)	0.036	0.060	0.552	1.036	0.922	1.165			
Baseline CGT	0.005	0.001	0.001	1.005	1.002	1.008			

Abbreviations: aDL, Activities of daily living; CGT, caregiver time; CI, confidence interval; GLM, generalized linear model; FIM, Functional Independence Measure; MNA‐SF, Mini Nutritional Assessment – Short Form; OR, odds ratio; SE, standard error; Sig, significance.

In the sensitivity analysis (supplement), the ITT‐analysis showed similar results as for the observed cases (Supplemental Table 1), 49 min in the IG and 71 min in the CG, although with some weaker statistical significance (*p* = 0.054; OR 0.476–1.007)).

## 4 DISCUSSION

### 4.1 The results

The major finding of the secondary analyses of the OPEN study is that a combined intervention of exercise and ONS may, beyond the positive nutritional and functional effects, also reduce CGT, especially for residents with cognitive disorders.

We observed that the baseline results of CGT in basic ADL support (83–105 min per day and resident) are in line with the figures seen in other studies where RUD has been used.^3^, ^5^ Next, we could partly confirm the hypotheses that the intervention would reduce CGT. There were small differences in terms of CGT between the IG and the CG when the dementia and somatic units were combined. However, when CGT was analyzed separately for the dementia and somatic units, a significantly reduced CGT emerged for the IG dementia units. The difference at the dementia units, about 28 min/day, and resident (which at a care unit with 20 residents corresponds to about 9 h per day of staff time) is not only statistically significant, but also clinically relevant. It could mean less stressful situations in caring and more time for communication and supporting the residents.

The ITT analysis in the sensitivity analysis resulted in a borderline significance with smaller time‐differences (22 instead of 28 min) at the dementia units. We nevertheless regard the ITT data as of the same clinical relevance as for the OC.

There is no simple explanation of the reduced CGT at the dementia units. The residents of the somatic units were significantly older and had, on average, higher risks for sarcopenia and frailty. They were also more dependent in ADL, and therefore their capacity to improve could be limited. Residents at the dementia units had lower cognitive function but significantly better ADL function at baseline, and perhaps a higher potential to improve their functional ability. A higher functional status was also associated with higher adherence to the intervention.^18^ In the staff interviews, it was reported that persons with better physical function either took responsibility to perform the intervention themselves or that it was easier for staff to support the person to complete the intervention.^21^ However, the qualitative studies were performed without taking account of whether the residents or staff resided or worked at dementia or somatic units, respectively, which hampers conclusions to be drawn from the two care forms separately.

Previous studies using RUD analyzing CGT have reported associations between higher dependency in ADL and increased CGT.^3^, ^5^, ^10^, ^13^ However, given all these uncertainties, the unit type differences must be judged cautiously, and further studies are needed addressing how cognitive status and competence at dementia units might influence the effects of physical exercise on ADL.

The interpretation of a reduction in CGT may be problematic. Is it good or bad that the residents need/receive less CGT? In the OPEN study, the intervention was delivered by the staff at each unit.^16^ It could be hypothesized that the responders became more independent in parts of their ADL functions and therefore needed less support from the staff in their daily care, which would decrease CGT. It could also be assumed that the intervention improved the communication and relationship between residents and staff, since the intervention was completed by the LTIC staff together with the residents and not by research staff.^16^ Such suggestions are supported by the results of the qualitative interview study with the residents.^19^ It could thus be hypothesized that the staff could reduce the support in the residents’ ADL as they became more independent and self‐supporting. The findings from the staff interviews, also align with this theory^21^ indicating that the staff adjusted the care and support to fit the resident's reduced needs, which also made the exercises more easily tolerated by the residents. This, in turn, tended to act as a staff motivator.

A negative interpretation could imply that the reduced CGT indicates neglect by the staff. However, this outcome appears unlikely since one of the key prerequisites for success enabling activities with person‐centered support is having a combined encouraging and empathic attitude.

The study period was 12 weeks. Other commonly used resources (such as hospitalizations) showed no statistical differences. Such resources are more inert to change and needs more time to response to an intervention (for example one year).

At the time when the OPEN study was planned, LTIC staff had more focus on fall prevention than physical function,^36^ whereas today the importance of physical activity (and ONS) for older frail persons are better recognized.^37^

Variations in staff density between the dementia and somatic units may impact the results of this study. When the OPEN study was conducted, nearly all dementia units in the Stockholm area had more nursing staff employed compared to somatic units. In a survey in 2013 by Stockholm City Council,^38^ the annual staffing proportion, as a general term for a mean staff assignment expressed as the staff‐to‐resident ratio, was 0.98 in somatic units compared with 1.06 in dementia units. Many dementia units, including the units where the OPEN study was conducted, had implemented educational activities for staff in dementia units based on guidelines for dementia care from the Swedish National Board of Health and Welfare launched which focused on person‐centered care.^39^

### 4.2 Methodological issues and limitations

Does the reduction in CGT indicate cost‐effectiveness in the dementia units? From a payer's viewpoint, a reduction in CGT may be regarded as beneficial. However, this question cannot be answered for two reasons. First, cost‐effectiveness statements need a relationship to an outcome, for example, function, QALYs (quality‐adjusted life‐years), and second, a reduction in CGT is only one part of a comprehensive resource. All other parts of the resource use showed non‐significant changes, and we have not assigned any costs to the intervention.

CGT was a secondary outcome in the OPEN study. The differences between the dementia and somatic units was also a finding from secondary analyses and not part of the original protocol. Thus, the results need to be assessed with caution and considered explorative and rather hypothesis‐generating. Prospective studies are needed for confirmation. In such study, CGT should be the primary outcome with a pre‐determined hypothesis and analysis plan. Although a significant difference was observed between the two types of units, the sample size was rather small. The overall difference between the IG and CG, about 12 min, was not significant. The rather large standard deviations of the CGT values, as well as the skewed data, indicate that larger samples are needed for the analysis of resource use in LTIC. The power calculation was based on the primary outcome, the CST. In general, data on resource use need larger sample sizes than clinical outcomes. However, it is also regarded as unethical to over‐power a trial. Since the sample size calculations were based on the primary outcome, it was a disadvantage in this secondary analysis.

Due to exclusion criteria, about one third of the residents were eligible for the study. Thus, our results are not generalizable to all residents in LTIC. Multiple testing was also conducted, which increases the risk for type I errors.

One plausible explanation for the differences in CGT between somatic and dementia units is that the Hawthorne effect^40^ was stronger at the somatic units, since residents there have better cognitive function, and thus better awareness of being observed even if they not have access to the intervention. Staff may also move between units in a way we don´t have control for. This may imply that the intervention effects at the somatic units can be under‐estimated.

To justify a causal relationship in an intervention, all factors that might impact results must be known and controlled for. In the OPEN study, the randomization was cluster‐based and not individual. This design was needed, but we are aware of that cluster‐randomization may include factors that are difficult to control for. Although it was not predefined to split the analyzes into dementia and somatic units, the cluster‐randomization did nevertheless include units that were labeled as dementia and somatic units. We did not assess different organizational factors, such as staff ratio, skill‐mix, previous educational activities or support from managers, or healthcare professionals and colleagues, at the somatic and dementia units. These organizational factors, such as higher levels of staff in dementia units compared with somatic units and new national guidelines including education for staff in dementia units,^39^ could also have positively influenced the effect of the combined intervention and thereby decreased the CGT in dementia units compared with somatic units. Future studies on the intervention's effects on CGT should include organizational factors. A further limitation is that we have no data on medication use or changes in medication during the intervention.

In conclusion, at LTIC dementia units, the OPEN intervention of combined daily sit‐to‐stand exercises and protein supplementation for 3 months resulted in a both statistically and clinically significant reduction, in CGT, which was not seen in the corresponding somatic units. The reason for this effect needs to be elaborated in further studies.

## CONFLICT OF INTEREST STATEMENT

The following authors declare no conflicts of interest (none): Gerd Faxén Irving, Erika Franzén, Helena Grönstedt, Sofia Vikström, Åke Seiger, Anne‐Marie Boström. Tommy Cederholm has previously received unconditional research funding as well as honoraria for lectures from the sponsoring agent (Nutricia). Anders Wimo is a copyright holder of the Resource Utilization in Dementia (RUD) instrument (part) but has not received any license fees for the use of RUD in this project. Author disclosures are available in the Supporting Information.

## CONSENT STATEMENT

All human subjects provided an informed consent before study inclusion, and in a few cases, strengthened by a legal representative.

## Supporting information



Supporting Information

Supporting Information
