# Supplementary material for: Impact of an exercise and nutrition program on caregiver time with residents in institutional care—A secondary analysis
Source: Alzheimers Dement. 2026 Feb 14;22(2):e71198. doi: 10.1002/alz.71198 (PMC12906372; doi:10.1002/alz.71198)
Supplement: Supplementary file 1 — Supporting Information [file ALZ-22-e71198-s001.docx]

Supplemental table S1. Sensitivity analysis of CGT. GLM-analyzis with ITT-approaches.

| **Parameter** | B | SE | Sig | OR | Lower 95% CI of OR | Upper 95% CI of OR | CGT at follow-up | Lower 95% CI | Upper 95% CI |
| --- | --- | --- | --- | --- | --- | --- | --- | --- | --- |
| ITT: imputation by Expectation-Maximization (EM) algorithm | | | | | | | | | |
| **All** | | | | | | | | | |
| Intervention group (n=60) | -0,12 | 0,13 | **0.38** | 0,89 | 0,68 | 1,16 | 61,9 | 51,3 | 74,7 |
| Control group (n=60) |  |  |  |  |  |  | 69,6 | 57,8 | 83,9 |
| **Dementia units** | | | | | | | | | |
| Intervention group (n=37) | -0.37 | 0.19 | **0.05** | 0.69 | 0.48 | 1.01 | 49.2 | 38.6 | 62.8 |
| Control group (n=29) |  |  |  |  |  |  | 71.1 | 53.9 | 93.7 |
| **Somatic units** | | | | | | | | | |
| Intervention group (n=23) | 0.14 | 0.21 | **0.51** | 1.14 | 0.76 | 1.72 | 81.8 | 60.4 | 110.9 |
| Control group (n=31) |  |  |  |  |  |  | 71.5 | 55.1 | 92.7 |

SE=Standard error, Sig=significance, OR=Odds Ratio, CI=Confidence interval.
